# Effects of Reduced Space Allowance and Heat Stress on Behavior and Eye Temperature in Unweaned Lambs: A Pilot Study

**DOI:** 10.3390/ani11123464

**Published:** 2021-12-05

**Authors:** Laura Menchetti, Leonardo Nanni Costa, Martina Zappaterra, Barbara Padalino

**Affiliations:** Department of Agricultural and Food Sciences, University of Bologna, Viale Fanin 46, I-40127 Bologna, Italy; laura.menchetti@unibo.it (L.M.); leonardo.nannicosta@unibo.it (L.N.C.); barbara.padalino@unibo.it (B.P.)

**Keywords:** transport, discomfort, unweaned lambs, space allowance, hot temperature, regulation

## Abstract

**Simple Summary:**

In Europe, young lambs can be transported long distances for slaughter. While transport is regulated by E.U. law, there is a lack of research investigating the optimal transport conditions specifically for young lambs. For example, while the regulations set a minimum space allowance for lambs above 26 kg, no minimum is specified for young lambs meaning they can be transported in overcrowded conditions. Further, while the temperature within the vehicle must be maintained between 5–30 °C, this is well above the 21 °C said to be the upper end of the comfortable range for lambs. This study aimed to investigate how variation in space allowance and temperature can affect the welfare of young lambs. Three groups of lambs were created where either the density of individuals (0.27 vs. 0.20 m^2^ per head) or the temperature range (12–18 °C vs. 19–30 °C) varied. Lambs housed in the higher density and heat stress groups showed more discomfort and higher body temperatures. In addition, lambs kept in the heat stress group lost weight over the course of the study, and showed signs of heat stress when the temperature exceeded 25 °C. These results indicate that the regulations for the transport of young lambs need to be refined.

**Abstract:**

Current European animal transportation law contains only a few and vague indications concerning how to move lambs of less than 26 kg. Moreover, little information is available in the literature about factors affecting these lambs’ welfare. We investigated the effect of space allowance and ambient temperature on the welfare of unweaned Lacaune lambs during a simulation of long-distance transportation (19 h). Three groups of lambs (*N* = 130) were housed in equally sized pens for 19 h, Control (C; *n* = 39; 0.27 m^2^ per head), Low Space Allowance (LSA; *n* = 52; 0.20 m^2^ per head), and Heat Stress (HS; *n* = 39; 0.27 m^2^ per head) groups. LSA lambs had lower space allowance than C but were tested at the same temperature, within their Thermoneutral zone (range = 12–18 °C). The HS lambs were, instead, subjected to higher temperatures (range = 19–30 °C). Scan sampling of behavior was conducted, eye temperature and body weight were also recorded. LSA and HS lambs showed more discomfort behaviors (*p* < 0.05) and higher eye temperatures (*p* < 0.001) compared to C lambs, while HS lambs additionally showed a decrease in body weight over the experimental period (*p* < 0.001). This study indicates that lower space allowances and higher temperatures impact negatively the welfare of lambs transported for slaughter suggesting that the regulation should be implemented taking these factors into account.

## 1. Introduction

Sheep farming is an important activity in the animal production sector of the Mediterranean countries [1] and worldwide [2]. The high plasticity of its breeding systems make sheep a livestock animal with a prominent socioeconomical role for marginal rural communities, but also for developed countries [3]. Worldwide, sheep breeding is used to obtain a wide range of products, including fiber, milk, and meat coming from adult and young animals (lambs). In Italy, the lamb market is characterized by a marked seasonality and a consumption that is traditionally rooted in Southern and Central Italy, with most of this meat being consumed during Christmas and Easter periods. According to the Italian Statistical Office (ISTAT), 2.3 million head of lambs were slaughtered in 2020, 15% of which came from other European countries, mainly Romania and Hungary [4], necessitating long transport of live animals.

In Europe, road transport of animals is regulated by EC Regulation No. 1/2005, but the maximum journey duration allowed varies depending on the animal species and category. For unweaned lambs, the maximum journey duration allowed is 19 h, comprised of 9 h of road transport, followed by a compulsory one-hour resting stop during which the animals must be fed without unloading, and then another 9 h of road transport. Vehicles must be equipped with onboard watering systems (i.e., water must be always available) and can have multiple adjustable levels. The minimum space allowance during transport allowed per head ranges from 0.20–0.30 m^2^ per head for sheep from 26-55 kg. However, for smaller lambs (<26 kg) an area of under 0.20 m^2^/animal may be provided (Chapter VII in Annex 1 of EC Regulation No. 1/2005) [5]. The unspecific regulations concerning lambs, therefore, allow transporters to load a large number of subjects per journey without considering the stress due to overcrowding. Padalino et al [6] highlighted that space allowance is a risk factor for the mortality and morbidity of lambs during long journeys.

The Farm Animal Welfare Council (FAWC) [7] indicated that a rough guide to obtain a minimum space allowance could be calculated by the formula A = 0.021 × BW^0.67^, where BW is live body weight (kg), A is the area covered by the subject (m^2^) and 0.021 is a value (k) that depends on several factors such as environmental conditions, age of the animals, and typical posture adopted by each species [8]. After revising several formulas, Petherick and Phillips [8] indicated the formula A = 0.027 × BW^0.66^ as the most adequate to allow all animals within a pen or a vehicle to be able to lie simultaneously, and suggested the formula A = 0.033 × BW^0.66^ as the best to minimize the risk of poor welfare and productivity during long term confinement. More recently, guidelines published in 2017 by the European Animal Transport Project [9] suggested a minimum space for lambs (approx. BW = 20 kg) of 0.27 m^2^, which, approximately, corresponds to a k of 0.037. The k value is however dependent also on the environmental variables and the animals’ ability to maintain thermoneutrality [10].

The thermal condition during transportation may indeed have different welfare impacts and heat stress is considered as a major cause of sheep mortality during live export [11,12]. Global warming is leading to increased frequencies of hot and muggy summer months, increasing the exposure of animals to environmental conditions that exceed the Upper Critical Temperature (UCT) of their Thermoneutral Zone (TNZ). TNZ is the range of ambient temperature within which there is a balance between body heat production and body heat loss [13]. The TNZ for sheep is reported to be between 12 and 27 °C by Marai et al. [14], but other authors suggest lower values (i.e., 5–25 °C) [15,16,17]. The UCT describes the point above which an animal must significantly increase their use of physiological mechanisms to prevent a rise in body temperature above normal and consequently travelling above UCT may severely compromise animal welfare [18]. If the coping mechanisms to maintain homeothermy are unsuccessful, the increase of body temperature leads to acute heat stress that, if prolonged, can result in heat stroke and death. UCT varies with species, age, live weight, health status, feed intake and genetic type. In small ruminants, panting, fast breathing, weakness, inability to stand, and an elevated rectal temperature are commonly used as Animal-Based Measures (ABMs) for heat stress [19]. Within TNZ, there is the thermal comfort zone which is where neither metabolic rate nor animal behavior are activated in any way to keep body temperature within the normal range [20]. Calculating the exact value of TNZ, comfort zone, and UCT is difficult and great variability exists in the literature depending on the age of the animal, sex, and whether animals are fleeced or shorn [14,15,16,17,21,22,23]. Due to the high variability, it is crucial to minimize thermal distress and to control environmental parameters inside the vehicle so that they suit the category of animals transported [24].

Space allowance should be increased when there is a risk of heat stress in transit because more space around the animal facilitates thermoregulation. However, the scientific literature lacks studies investigating UCT and minimum space allowance in lambs. The hypothesis of this preliminary study was that unweaned lambs (BW < 26 kg) kept at the space allowance suggested by the Consortium of the Animal Transport Guides Project (ATG, 0.27 m^2^) [9] would show less discomfort (more resting behavior and less stress-related behaviors) than those kept at the lower space allowance (0.20 m^2^; [5]) currently permitted by the EC Regulation 1/2005. However, high temperatures also influence these behaviors reducing the beneficial effects of the larger space allowance. Thus, we also hypothesized that even lambs kept with a space allowance of 0.27 m^2^ would show discomfort-related behaviors when exposed to temperatures above the comfort thermal zone of the TNZ. The aim of this preliminary study was therefore to document the effect of space allowance and temperature on non-invasive ABMs of welfare, such activity, lying position, social behavior, panting, and eye temperature (ET), in unweaned lambs in a simulation of a long journey. 

## 2. Materials and Methods

The study was carried out at the Fattorie Rabboni e Zanetti, Meldola, Emilia-Romagna, Italy. Experimental protocols were approved by the Animal Experimentation Ethics Committee of the University of Bologna (Prot. n. 0002777, 15 April 2021).

### 2.1. Experimental Design: Animals and Housing Conditions

**Figure 1:**
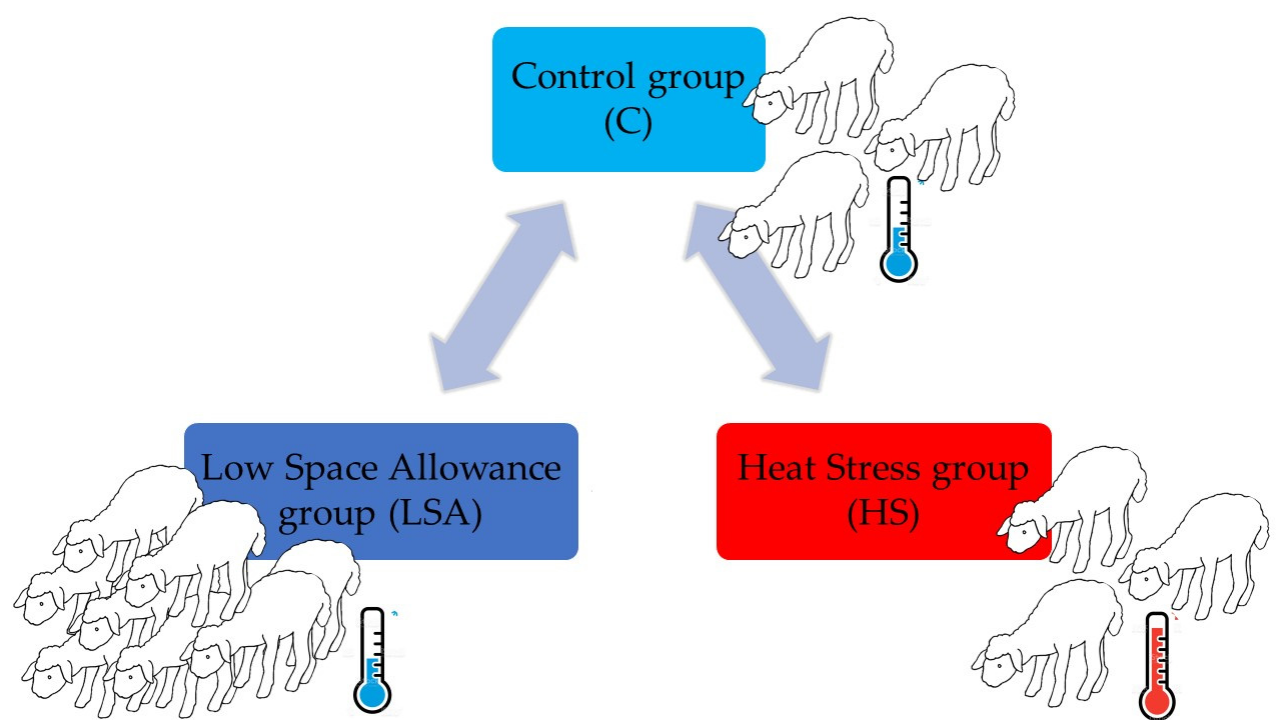
Representation of the experimental groups. Three groups of lambs were used. Lambs of the Control group (C) were kept at a space allowance of 0.27 m^2^ and temperatures within the Thermoneutral zone. Lambs of the Low Space Allowance group (LSA) were kept at the same temperatures but had a space allowance of 0.20 m^2^. Finally, lambs of the Heat Stress group (HS) were kept at a space allowance of 0.27 m^2^ but above the thermal comfort zone.

In this case, 130 Lacaune unweaned lambs of both genders, approximately 60 days old, with an average live body weight of 22.3 (±3.8) kg, were used in two phases of the same experiment. The first phase evaluated the effect of space allowance on lamb behavioral and physiological parameters. It was conducted during the spring period (the 15th and the 16th of April 2021; environmental indoor temperature: mean = 15 °C, range: 12–18 °C; environmental outdoor temperature range = 4–14 °C) and involved 91 lambs. Lambs were randomly assigned to two groups and arranged in two equal indoor pens (2.97 m × 3.5 m) with different space allowance: 0.27 m^2^/lamb (Control group, C, *n* = 39) and 0.20 m^2^/lamb (Low Space Allowance group, LSA, *n* = 52), which correspond with the space allowance recommended by ATG and the upper limit of Regulation 1/2005 for this animal category, respectively. The second phase investigated whether the environmental temperature influenced the studied ABMs of the lambs kept at the widest space allowance (0.27 m^2^/lamb). It was conducted during late-spring period (the 15th and the 16th of June 2021; environmental indoor temperature: mean = 25 °C, range = 19–30 °C, Supplementary Figure S1; environmental outdoor temperature range: 14–30 °C) and involved 39 lambs arranged in the same indoor pen (2.97 m × 3.5 m) used in phase 1 and with a space allowance of 0.27 m^2^/lamb. In this group (Heat Stress, HS), therefore, lambs had the same space allowance as the C group but were exposed to higher temperatures and humidity (Figure 1).

Before the start of each simulation (T_start_), lambs were identified by numbers painted on their sides and rump (using a washable spray for animal marking) and weighed individually using an electric scale (C.M.S. Bilance, Modena, Italy). The indoor temperature and relative humidity were continuously monitored using weather stations (Kestrel 4000, Nielsen-Kellerman Company, Boothwyn, PA, USA) positioned at about 1.50 m high in order to avoid contact with the animals. The experimental conditions of each group, including environmental temperature and humidity, mean body weight (BW) of lambs, animal density and their corresponding k coefficient, are summarized in Table 1.

**Figure 2:**
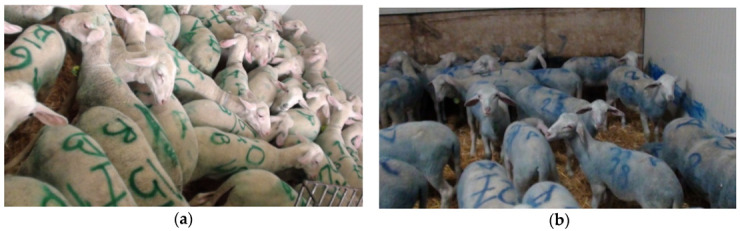
Group of lambs kept at 20 cm^2^/animal (Low Space Allowance, LSA; panel (**a**) and Group of lambs kept at 27 cm^2^/animal (Control, C; panel (**b**) during a long journey simulation.

The lambs were kept in their pens for 19 h to simulate the maximum allowed travel duration (from 1:30 pm to 8:30 am the following day) and fed milk replacer (Emme Erre Capri Ovi, Tredi Italia, Cremona, Italy) using four automated milk feeding systems (Lupetta, Smart Feeding System^®^, Crema, Italy) which supplied 3 teats per pen. A total of 115, 175, and 131 L of milk was suckled by the lambs of C, LSA, and HS groups, respectively, which corresponds to an average of 2.95, 3.37, and 3.36 L per lamb. Additionally, lambs were given access to a mixed feed for one hour (from 10:30 pm to 11:30 pm) consisting of commercial pellets and graminacaea hay, to simulate what happens in a journey (journey time: 9 h travel, 1 h resting with feeding, 9 h travel). All pens were in the same building, had straw bedding, were separated by non-transparent plastic panels, and had a plastic trough to provide water *ad libitum*. Pens were artificially illuminated day and night, and no variations of the lighting were caused by the experiment (Figure 2).

At the end of each simulation (T_end_), lambs were weighed again, and the difference compared to the initial weight was calculated (∆BW = BW at T_end_ − BW at T_start_).

### 2.2. Behavioral Observations

The lambs were recorded by a security camera system (TechView DVR Kit, Model Number QV-3034, Petaling Jaya, Selangor, Malaysia) placed in each box. The camera was placed 2.5 m above the ground and oriented towards the feeding and drinking area, allowing the observation of the whole pen except for a blind spot of about 3 m^2^. The animals were video-recorded during the entire experimental period (i.e., 19 h). All videos were simultaneously analyzed by two trained observers using a scan sampling method and the ethogram shown in Table 2. Behavior was analyzed every 5 min (12 scans per h) over the 19-h period with a total of 228 scan samples per box. Each scan was viewed a first time to define the states of each lamb (behaviors included in the “Activity and lying position” and “Panting” categories) and a second time using a behavioral sampling ethogram to record the frequency of the behavioral events that occurred in a time window of 30 s (behaviors included in the “Interactions” and “Other behaviors” categories). As the camera could frame about two-thirds of each pen and not all animals were visible in each scan, the results were expressed as a percentage of animals engaging the behavior in relation to the total number of visible animals at each scan (for the states) or percentage of events occurring in 30 s in relation to the total number of visible animals (for the events). 

Then, the proportions of all behaviors indicating both the sternal and lateral decubitus and standing included in the “Activity and lying position” category were summed to create two new variables named Total Recumbency and Total Standing, respectively. Furthermore, for the Total recumbency, the total proportion of animals with head held high and head down was calculated creating two other variables called “Head-up recumbency” and “Head-down recumbency”, respectively.

### 2.3. Eye Temperature (ET)

**Figure 3:**
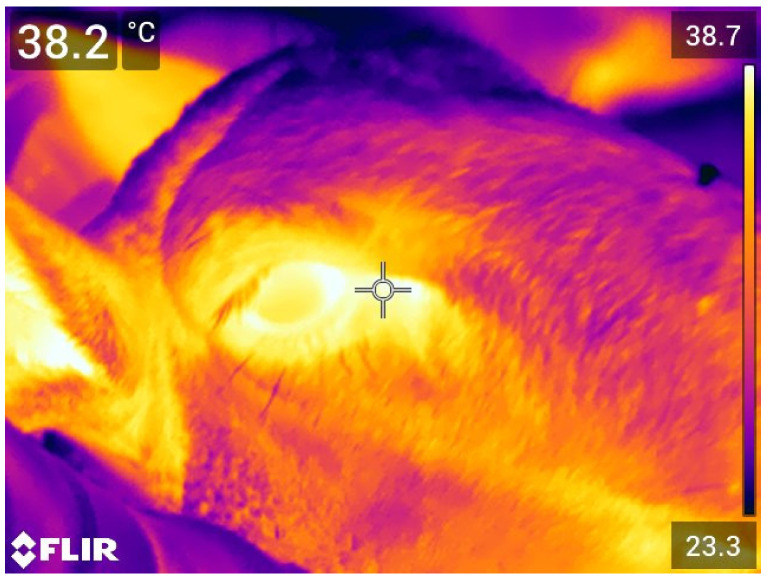
Eye temperature taken using a portable camera (FLIR E76 24°; FLIR Systems AB, Danderyd, Sweden) during a simulation of a long journey in lambs kept at two different space allowance.

At the end of each simulation (T_end_), infrared thermography images of the lamb heads were taken using a portable camera (FLIR E76 24°; FLIR Systems AB, Danderyd, Sweden) (Figure 3). The resolution of the camera was 320 × 240 pixels, and the accuracy was ±2°C or ±2% at environmental temperatures ranging from 15 to 35°C. The camera was calibrated using the environmental temperature and relative humidity recorded by the weather station (Kestrel 4000, Nielsen-Kellerman Company, Boothwyn, PA, USA) in the pen. The camera was positioned at 90° to the sagittal plane and at a distance of approximately 0.40 m from the lamb’s right side. The images were analyzed using the FLIR Tools^®^ software (FLIR Systems AB, Danderyd, Sweden) focusing on the lacrimal caruncle of the right eye to determine maximum temperature (ET), as previously reported [32,33,34]. 

### 2.4. Statistical Analysis 

Descriptive statistic was used to present the data by means, standard error (SE), medians (M) and interquartile ranges (IQR). Diagnostic graphs, Shapiro and Levene’s tests were used to verify the assumptions and identify outliers. Welch’s ANOVA was used to evaluate the group effect on ∆BW and ET as these variables did not meet the homoscedasticity assumption. Dunnet post hoc test was then performed selecting the C group as the control reference. Generalized linear models were used to evaluate the effect of the group on the behavioral variables, with parameters estimated by generalized estimation equations. Negative binomial distribution and logarithmic link function were set. Models evaluated the main effect of the group with the time (in hours) included as a covariate. Scans were included in the models as the within-subject variable. Simple contrasts were planned where the C group was treated as the reference category. Finally, Spearman’s rho coefficient (ρ) was used to evaluate the associations between the proportion of panting lambs and environmental parameters. The correlation was considered weak if ρ < │0.4│, moderate if │0.4│ ≤ ρ < │0.7│, and strong if ρ ≥ │0.7│. Analyses were performed using SPSS version 25 (SPSS Inc., Chicago, IL, USA), setting significance at 0.05. GraphPad Prism, version 7.0 (GraphPad Software, San Diego, CA, USA) was also used for data visualization.

## 3. Results

At the end of the trail, no lambs died or showed clinical signs such as nasal discharge or diarrhea.

### 3.1. Body Weight Changes 

**Figure 4:**
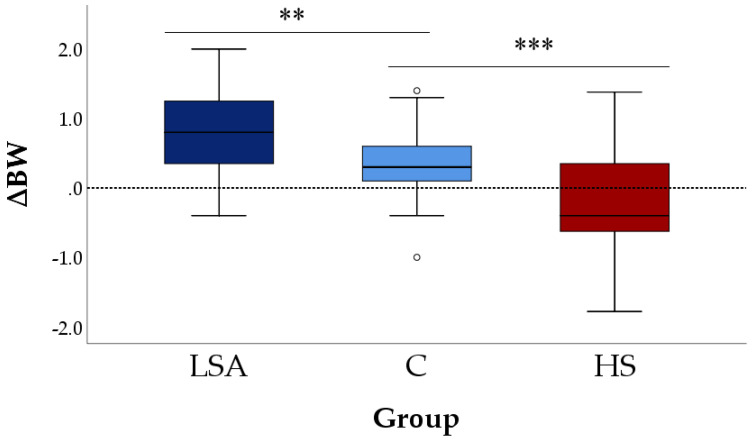
Changes in body weight (∆BW) during the simulations in the three groups. Each box plot shows the median, interquartile ranges, low and high extreme values (one and a half times the interquartile range if outliers are present); dots show outliers. LSA = Low Space Allowance group (0.20 m^2^/lamb in TNZ), C = Control group (0.27 m^2/^lamb in TNZ), HS = Heat Stress group (0.27 m^2^/lamb above the comfort zone of the TNZ). *** *p* < 0.001, ** *p* < 0.01 compared to the C group (Dunnet post hoc test).

The delta in body weight (∆BW) had positive medians in both C and LSA groups while it had a negative median in the HS group (Figure 4) indicating that, regardless of the space allowance, most lambs gained weight while were kept in the comfort zone of the TNZ while lost weight when exposed to hot temperature (*p* ≤ 0.001).

### 3.2. Activity and Lying Position 

**Figure 5:**
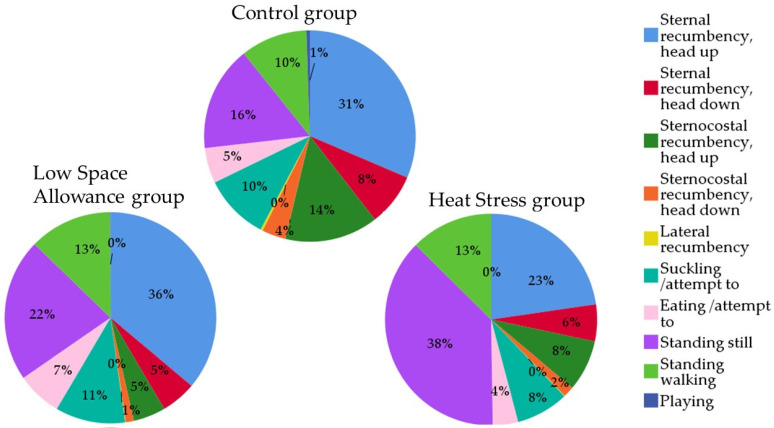
Relative proportions of the mutually exclusive behaviors indicating lamb activities and lying position in the three groups (mean percentage of visible animals recorded at 5 min interval over a 19-h period).

Figure 5 and Table S1 show the behavioral states included in the Activity and lying position category (mutually exclusive). A significant effect of space allowance and temperature was found for all these variables and pairwise comparisons showed several significant differences in both LSA and HS groups when compared to the C group (lambs kept at 0.27 m^2^ in TNZ). In particular, the reduction in space allowance (LSA group) increased the proportion of animals lying in sternal recumbency with head up, standing still, and walking while decreased the proportion of animals lying in sternal recumbency with head down, sternocostal (both with the head up and down), and lateral recumbency. Group LSA also showed a higher proportion of suckling/attempt to suckle and eating/attempt to eat lambs than C (*p* < 0.001 for all). The increase in temperatures (HS) resulted instead in a reduction of suckling and eating animals while the proportion of standing still animals was more than doubled compared to C (*p* < 0.001 for all). Finally, an appreciable value for the “Playing” variable was only found in C group (0.54 ± 0.14%), very low percentages were found in LSA (0.06 ± 0.04%) while the lambs of HS group did not play at all.

**Figure 6:**
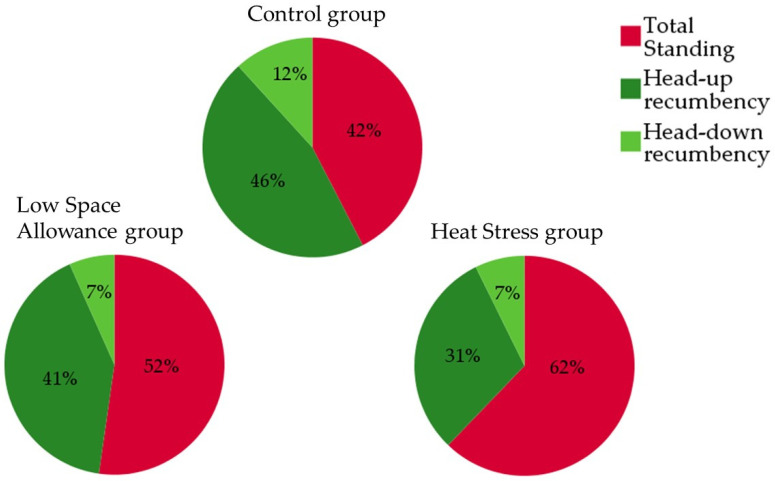
Relative proportions of Total Standing and Total Recumbency, categorized as Head-up and Head-down recumbency, in the three groups (mean percentage of visible animals collected every 5 min over a 19-h observation period).

The variables obtained by combining the behaviors of the Activity and lying position category (i.e., Total Standing, Head-up recumbency, and Head-down recumbency) are shown in Figure 6. Both low space allowance and heat stress reduced the proportion of lying animals compared with the C group as well as the head-down position. More than half of the lambs in the C group were indeed lying (57.84 ± 1.82, 47.76 ± 2.02, and 37.93 ± 1.84% of lying animals for group C, LSA, and HS, respectively; *p* < 0.001), and more than 10% of them had their heads down (11.74 ± 0.02, 6.58 ± 0.01, and 7.39 ± 0.01% of lying animals with head down for group C, LSA, and HS, respectively; *p* < 0.001).

### 3.3. Other Behaviors and Panting

**Figure 7:**
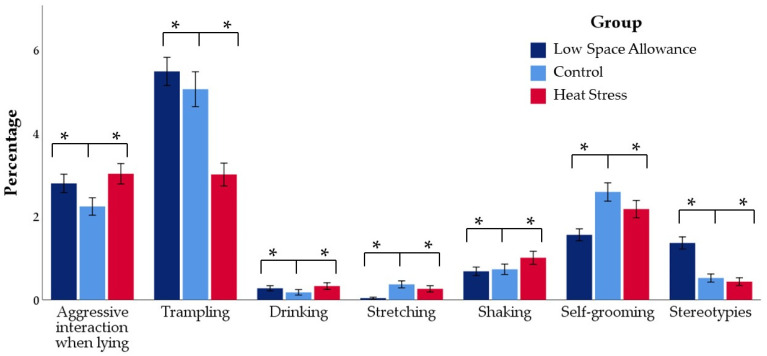
Relative proportions of the main behavioral variables included in the Interactions and Other behaviors categories (not mutually exclusive) in the three groups (mean percentage of events in relation to the number of visible animals collected every 5 min over a 19-h observation period). * *p* < 0.05 compared to the Control group.

Figure 7 shows the relative frequency of the main behavioral events included in the “Interactions” and “Other behaviors” categories while all variables are listed in Table S2. Results of pairwise comparisons are also presented where the group of lambs kept at 0.27 m^2^ evaluated within TNZ (C group) was treated as the reference category. Low space allowance (LSA group) and hot temperature (HS group) resulted in higher relative frequencies of aggressive interactions when lying but lower relative frequencies of aggressive interactions when active (8.63 ± 0.78, 8.10 ± 0.56, and 7.43 ± 0.77% for C, LSA, and HS, respectively; *p* < 0.001). Moreover, LSA group showed a higher relative frequency of trampling and stereotypies while the HS group showed higher percentages of affiliative behavior (5.96 ± 0.38, 3.51 ± 0.23%, 6.64 ± 0.37% for C, LSA, and HS, respectively), drinking, and shaking than C (*p* < 0.001 for all). Finally, the frequencies of the stretching and self-grooming were higher in the C group, compared both to LSA and HS groups (*p* < 0.001 for all).

**Figure 8:**
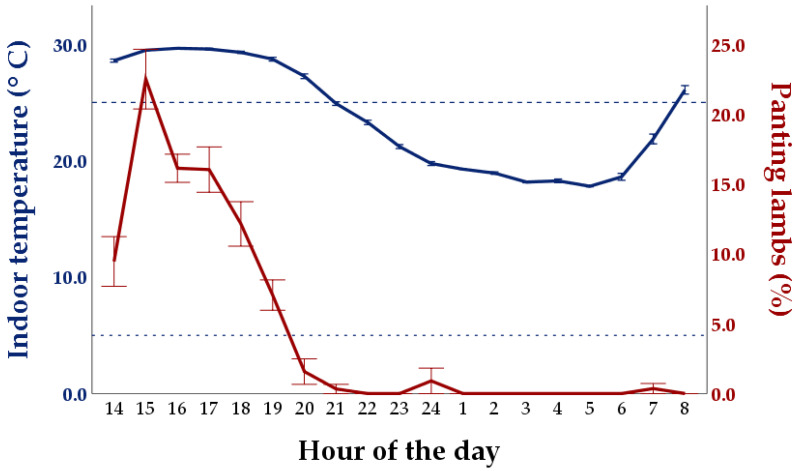
Changes in the indoor temperature and proportion of visible animals panting during the simulation conducted during the late-spring period (Heat stress group). No panting animals were observed in the simulations conducted in the Thermoneutral zone (Control and Low Space Allowance groups). Values are means±standard errors. The dotted lines indicate the thresholds of the Thermoneutral zone (i.e., 5–25 °C; [16]).

**Figure 9:**
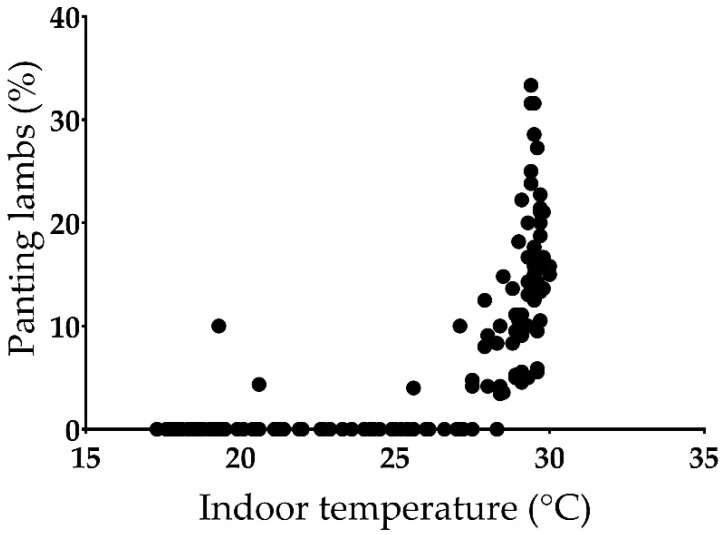
Scatter plot showing the relationship between the proportion of panting animals and indoor temperature for the simulation conducted during the late-spring period (Heat stress group). No panting animals were observed in the simulations conducted during the Thermoneutral zone (Control and Low Space Allowance groups).

Panting was only observed in the group evaluated outside the comfort zone of the TNZ (HS group: 4.57 ± 0.51%), with a pattern that followed the variation of the indoor temperature (Figure 8). A strong positive correlation between the percentage of panting animals and temperature was indeed found (ρ = 0.802, *p* < 0.01; Figure 9). The percentage of panting animals also showed a negative correlation with relative humidity (ρ = −0.798, *p* < 0.01). The latter variable had an almost mirror-like trend with respect to temperature (Figure S1) and the two variables were negatively related to each other (ρ = −0.961, *p* < 0.01).

### 3.4. Eye Temperature (ET)

**Figure 10:**
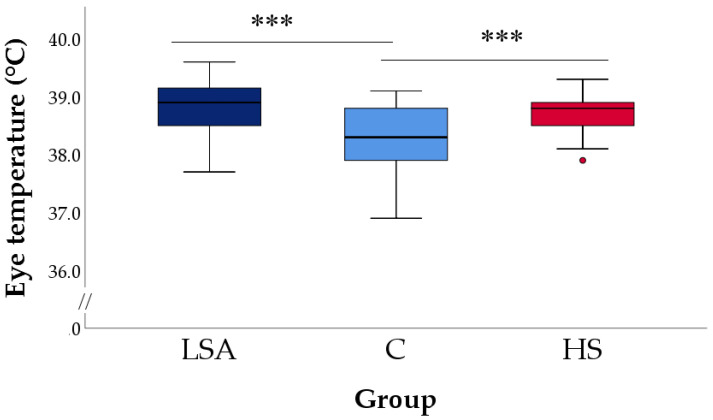
Eye temperature collected at the end of the simulation in lambs of the three groups. Each box plot shows the median, interquartile ranges, low and high extreme values (one and a half times the interquartile range if outliers are present); the dot shows an outlier. LSA = Low Space Allowance group (0.20 m^2^/lamb in TNZ), C = Control group (0.27 m^2/^lamb in TNZ), HS = Heat Stress group (0.27 m^2^/lamb above the TNZ). *** *p* < 0.001 compared to the C group (Dunnet post hoc test).

Figure 10 shows the box plots for the ET registered at the end of the simulation in all lambs. The lambs of the C group had a mean value of 38.24°C (±0.10 °C), which was statistically lower than those recorded in the other groups at the end of the journey simulation (LSA: 38.84 ± 0.09 °C; HS: 38.71 ± 0.05 °C; *p* < 0.001). 

## 4. Discussion

This preliminary study documented the effect of space allowance and temperature on the behaviors and ET in unweaned lambs of about 22 kg live weight during 19 h confinement, simulating a long journey for this animal category. Lambs kept with the lowest space allowance and those at the highest temperature showed significantly higher ET, and more signs of discomfort (such as more active behaviors and aggressiveness, fewer resting behaviors) than lambs kept at 0.27 m^2^/lamb within their thermal comfort zone (C group). Our data supported the hypothesis that the space allowance of 0.20 m^2^/lamb allowed by Regulation (EC) No 1/2005 is not sufficient to protect their welfare. An increased proportion of animals showing ABMs indicating discomfort were also found in lambs in HS group, suggesting that the space allowance of 0.27 m^2^/lamb may be not sufficient for lambs transported outside their thermal comfort zone. Our findings suggest that the current minimum space allowance reported in the EC 1/2005 should be revised for safeguarding the welfare of lambs during long journeys.

The proportion of animals standing was significantly higher in LSA than in C group. Moreover, LSA animals rested longer in sternocostal than lateral position keeping their head up. Although sheep and other herbivorous livestock can rest even while standing, studies have shown these animals have brain patterns that suggest a deeper sleep while resting in lying positions [35]. Thus, the change in time spent resting and the type of recumbency observed in the present study may have lowered the quality of resting time in LSA lambs. The fact that such animals experienced shallower sleep also appears to be supported by the decreased proportion of lambs stretching in this group compared to C. Stretching (referred also as pandiculation) is a behavioral expression found in several mammals that have been suggested as a way to reverse the muscular atonia during sleep and restore homeostatic functions during the transition between sleep and wake phase [36]. Stretching is also an expression of well-being [37,38]. The reduction of stretching frequency and the increased proportion of standing in the LSA group supports that providing 0.20 m^2^/lamb is not enough to enable lambs to rest in sternocostal recumbency and thus possibly impairing the normal sleep-wake cycle. 

Lambs in the LSA group also suffered more aggressions when lying compared to the C group. This increased aggressivity is in agreement with results in cattle [39], pigs [40], and camels [41,42] kept at limited space allowances. Although aggressive behaviors have been rarely observed among female sheep kept on pasture [43], limited resources such as restricted area shelter have been found to enhance aggressive behaviors also in sheep [30,44,45]. The lack of sufficient space allowance in the LSA group is also proved by the significantly higher proportion of animals trampling other lying lambs, in an attempt to reach a resting space, the water trough, or the automatic milking feeder. Trampling was not necessarily an aggressive attitude but could still result in discomfort and pain for lying lambs. Trampling and aggressions could indeed cause wounds and bruises, compromising not only animal welfare but also the final meat quality [46,47]. Aggressiveness is an expression of competition addressed towards gaining dominance on a resource with an intrinsic value for that particular animal species [48]. In previous studies, sheep have been shown to prefer lying next to a wall when kept in pens [49,50]. This would suggest that the increased aggressive behaviors displayed by lambs in the LSA group may be triggered by the attempt to gain the possibility of lying in preferred spaces. This result reinforces the observation that providing lambs with a space allowance of 0.20 m^2^/lamb is not sufficient to permit all lambs to accommodate simultaneously on their preferred spot. This point is of major importance in sheep as this species has been shown to follow synchronous resting and activity patterns [51]. This synchronization declines as sheep are kept in larger size groups and with lower perimeter space allowance per ewe [52] or at higher stocking density [53]. In particular, Jørgensen et al observed that this loss in the synchronization patterns was mainly due to low-ranked animals, which decreased substantially the time spent resting and thus experienced most of the distress [52], as reported in goats [54].

Stereotypic behaviors were more frequent in LSA lambs. Stereotypic behaviors are expressed by animals in stressful situations and/or not able to express species-related behavioral repertoires [55]. Stereotypies are quite infrequent in grazing sheep but have been recorded in confined sheep [49,56] and lambs [26]. The latter showed in particular oral stereotypies, such as licking and suckling of objects in the pen, when offered short-sized straw [26]. Literature lacks studies concerning space allowance and stereotypic behaviors in unweaned lambs. The present study is, therefore, the first indicating that limited space allowances prolonged for 19 h may trigger states of distress causing increased stereotypies in unweaned lambs, such as bar and wall licking.

In addition to low space allowances, high ambient temperature resulted to strongly influence the behavioral repertoire of the tested lambs. Lambs kept in the HS group spent more time standing still, while reducing other activities such as trampling, playing, self-grooming, and stretching. The observed effects are in line with behavioral and physiological coping mechanisms aimed to eliminate additional heat load or reduce heat production in animals subjected to heat stress. Behavioral plasticity in heat stress conditions leads other ruminant species to increase standing time and decrease activity and movement [42,57,58]. These changes are aimed to improve cooling, by decreasing heat generation from muscle and increasing the radiating body surface area exposed to air movement [57]. The increased time spent standing still to dissipate heat load may have disrupted the normal sleep-wake cycle and shallower sleep also in HS lambs. Heat load and extended periods of prolonged standing have been argued to cause frustration and aggressive behaviors in donkeys [59], pigs [60], and cows [39]. Likewise, we noticed an increased proportion of aggressive behaviors in HS lambs. The HS lambs spent also more time drinking. Water and liquids intake has been proved to increase significantly in heat stressed animals [61] as increased panting and sweating use water vapor to dissipate excessive heat load, causing loss of body water and increasing water requirements [62]. However, all animals considered in the present study consumed milk replacer if provided. This suggests that unweaned lambs should be provided with milk when transported for long periods, and future recommendations for more welfare-friendly animal transportation should take into account also the possibility to supplement milk replacer to unweaned animals during long journey transport. Body shaking was also more expressed in the HS group. In sheep, this type of behavior is more common among young individuals [63], but its exact reason remains quite unknown. In dogs, body shaking without an apparent cause is a behavior suggestive of nervousness [64]. We are not able to fully explain the increased frequency of body shaking in the HS lambs; it may have a pattern similar to that observed in dogs or be a way to cope with heat stress attempting to increase heat dissipation. Environmental conditions were thus proved to be an important factor in determining discomfort during the tested confinement of 19 h, to the extent that when temperatures were above TNZ, even a space allowance of 0.27 m^2^/lamb was associated with behavioral expressions suggestive of altered sleep-wake cycles and increased frustration.

Lambs kept in LSA and HS groups also displayed significantly higher ET. This result agrees with the scientific literature, as increased core body and skin temperatures have been associated with the physiological responses activated in animals by hot ambient temperatures or stressful situations, such as overcrowding and competition [65]. The higher ET observed in the LSA group may thus originate from the combined effect of overcrowding and stress caused by increased competition to gain the benefits of limited resources (such as resting space and the teats of the automatic milking feeder). The higher ET observed in HS group is instead consistent with the physiological responses carried out by ruminants attempting to cope with ambient temperature exceeding the TNZ. In homeothermic species, the hypothalamus coordinates some of those responses, seeking heat dissipation through increased peripheral circulation, vasodilation, and sweating [66,67]. Together with vasodilation and sweating, panting is another cooling mechanism engaged when animals’ core/skin temperatures increase [66]. While in LSA and C groups panting was never observed, the proportion of panting lambs peaked to 30% when the ambient temperature rose above 25 °C. The latter has been indicated as the upper threshold of the TNZ in lactating ewes [16], and our results seem to confirm that at 25–27 °C the physiological mechanisms related to heat dissipation are activated in Lacaune lambs of about 22 kg live weight. As previously reported, UCT may greatly vary depending on sheep breed, age, weight, and nutritional state, and the temperatures we found may not be valid in animals of other ages, reared in different environmental conditions, and belonging to other breeds. In addition, the significant increase in the ET found in LSA and HS lambs confirms IRT as a possible viable tool to measure stress in livestock animals [32,68,69,70]. In agreement with EFSA opinion on pigs [71], ET may thus be considered a reliable ABM to assess in a non-invasive and effective way animal welfare consequences due to heat stress and impeded movement in lambs.

Taken together, the ABMs used in the present study suggest that lambs kept in LSA and HS groups were in distress and showed an increased proportion of behaviors associated with a negative welfare status. In accordance with that interpretation, positive indicators of welfare, such as playing and self-grooming, were noticed exclusively, or mainly, in the C group. Although being still poorly investigated, play-like behaviors are commonly considered a marker of positive animal welfare. Different animal species have different types of play behaviors, but several studies have confirmed that play is exclusively, or mostly, expressed by animals in good health and well-fed [72,73]. Play is therefore considered to actively promote positive welfare states in animals [31]. While the play is commonly reported to be a positive welfare indicator, the positive value of self-grooming is under debate and may vary depending on environmental conditions [74,75]. However, self-grooming has a programmed innate root, whose expression in small ruminants is highly dependent on age, with young individuals showing more self-grooming than adult ones [76]. In this view, self-grooming must be considered a positive behavior that is naturally more expressed in young subjects kept in environmental conditions permitting a wider range of emotional and behavioral expressions. To date, the expression of positive welfare states is widely suggested as an important point to be included during animal welfare assessment, as it provides not only an indication of animals being in good health status, but also experiencing positive emotions and well-rounded lives [31,77,78]. In view of this, the positive welfare indicators found in the C group reinforce the evidence that the space allowances and environmental conditions experienced by lambs in LSA and HS groups were not sufficient to sustain an acceptable welfare condition in lambs of about 22 kg live weight during confinement, similar to a long journey of 19-h.

Our findings should be interpreted with caution because this was a preliminary study with several limitations. The lack of replicate groups may have lowered the statistical power of the study. Furthermore, this pilot study was conducted in field, using unweaned lambs reared for the stock replacement in a commercial farm. For ethical restraints and to avoid possible health problems related to long fasting periods, lambs were given free access to milk, using automatic milking systems. During real transport conditions, animals are subjected to long fasting periods, vibrations and truck movements, and to other stressors, such as loading and unloading operations. All these environmental conditions have not been tested in the present study, and further analyses should be carried to evaluate the combined effects of these variables and space allowance on lambs’ behavioral repertoire and stress-related physiological response. In addition, operating in an in-field situation and using the equipment present on the farm, in the LSA group we had to reduce the space allowance per head by increasing the number of animals in the pen, which could also have had an effect on dominance relationships within the group and on the increased delta in body weight (∆BW) noticed in the LSA compared to C group. Notwithstanding these limitations, this is the first study to relate behavioral changes, ET, different space allowance, and changes in ambient temperature in unweaned lambs during the confinement of 19 h, simulating long journeys for these animals. We also observed that the unweaned lambs consumed high amounts of milk replacer during the 19 h of the study. This indicates that even when transported, these animals would likely consume milk. It would therefore be important to allow such supplementation in transit. This would allow better animal welfare and prevent excessive weight losses during long journey transport. The results obtained in the present study could be useful for suggesting possible recommendations, such as minimum space allowance, maximal effective temperature and the possibility to offer milk replacement, to be included in the revised European code of animal transportation as well as appropriate ABMs to assess the welfare of lambs.

## 5. Conclusions

Overall, this pilot study suggested using reliable ABMs that the maximum space allowance of 0.20 m^2^/lamb recommended in the current EC 1/2005 for lambs < 26 kg live weight is not enough to protect their welfare during transportation. The space allowance of 0.27 m^2^/lamb proposed by the Consortium of the Animal Transport was instead more acceptable when lambs were kept during their thermal comfort zone. Lambs in the latter conditions indeed expressed more comfort-related behaviors and fewer signs of distress. This space allowance was however associated with discomfort in lambs when ambient temperatures rise above TNZ. Replications and new trials during real transportations should confirm these findings. Further studies are also needed to establish whether greater space allowances may mitigate the effect of heat stress while travelling on lambs’ welfare. Anyway, the selected ABMs, including both physiological and behavioral measurements as well as positive and negative states, seem appropriate indicators of welfare consequences while the results could provide input for the implementation of the regulation.

## Figures and Tables

**Table 1:** Environment conditions, space allowance, mean body weight (BW), animal density and k coefficient of the three experimental groups.

Group	Temperature (°C), Mean and Range	Humidity (%), Mean and Range	N° Lamb/Pen	Space Allowance (m^2^/Lamb)	Mean BW ± SD (kg)	Animal Density (kg/m^2^)	Coefficient k
C	15 (12–18)	51(40–60)	39	0.27	22.79 ± 3.73	85.49	0.033
LSA	15 (12–18)	51(40–60)	52	0.20	21.18 ± 3.31	105.9	0.026
HS	25 (19–30)	65 (44–80)	39	0.27	23.46 ± 4.08	88.00	0.033

C = Control, LSA = Low Space Allowance, HS = Heat stress; BW = Body Weight, SD = Standard Deviation, k = constant of the allometric equation [8].

**Table 2:** Ethogram used to analyze the behavior in lambs using a scan sampling every 5 min over a period of 19-h.

Category	Behavior	Description
Activity and lying position^1^	Sternal recumbency, head up	Lamb is lying with the majority of body weight on the sternum; all limbs are under the body [25] and the head is up (above the height at the withers)
	Sternal recumbency, head down	Lamb is lying with the majority of body weight on the sternum; all limbs are under the body [25] and the head is down (at or below the height at the withers)
	Sternocostal recumbency, head up	Lamb is lying with the majority of body weight on the sternum but at least one hind limb is on one side, bent but visible and the head is up (above the height at the withers)
	Sternocostal recumbency, head down	Lamb is lying with the majority of body weight on the sternum but at least one hind limb is on one side, bent but visible and the head is down (at or below the height at the withers)
	Lateral recumbency, head down	Lamb is lying with the majority of body weight on the left or right side with all limbs outstretched to respective side [25], head and neck are extended on the floor
	Standing, suckling/attempt to	Lamb standing on all four legs, while milk feeding or trying to reach a teat
	Standing, eating/attempt to	Lamb standing on all four legs, searching for forage straw in the bedding or in the fodder rake (when it was offered), chewing and eating it [26]
	Standing, still	Lamb standing on all four legs, without moving [26], far from the milk station
	Standing, walking	Lamb stands on four legs but it moves around [26]
	Standing, Playing	Lamb shows any locomotory play or play fighting [27]
Panting^1^	No	No panting, normal respiration (panting score = 0) [28]
	Yes	Lamb shows a high respiration rate, it appears to “breath” from its flanks; the mouth could be closed (first phase, panting score = 1–2) or open (second phase, panting score > 2) [28,29]
Interactions	Aggressive interaction when lying	Lamb tramples with its front legs or pushes with its head another animal which is resting [30]
	Aggressive interactions when being active	Lamb pushes another animal with its head or other parts of its body or mount another active ewe (which is standing, eating, or moving) [30], often to reach the milk station
	Affiliative behavior [31]	Lamb interacts with another in a nonaggressive way (sniffing, licking, or allogrooming)
Other behaviors	Trampling	Lamb climbs over another lying animal trampling it, in a nonaggressive way, often having no alternative ways
	Drinking	Lamb drinks water from the drinker [26]
	Self-grooming	Lamb licks itself or lamb scratches itself against pen equipment
	Stretching	Lamb extends/stretches part or all of the body
	Shaking	Lamb performs quick sudden movements of the head or whole body
	Stereotypies	Lamb performing a frequent and non-functional oral manipulation (licking) of object such as pen bars, walls, fodder rake [26]

^1^ mutually exclusive behaviors.

## Data Availability

Data are available on request.

## Supplementary Materials

The following are available online at https://www.mdpi.com/article/10.3390/ani11123464/s1, Table S1. Effect of group and time on behavioral variables included in the Activity and lying position category. Values are means and standard errors of the proportion of visible animals engaging each behavior collected every 5 min over a 19-h observation period (228 scan samples per group). C = Control group, LSA = Low Space Allowance group, HS= Heat Stress group. Asterisks indicate significant differences compared to C group (*p* < 0.05). Table S2. Effect of group and time on behavioral variables included in the Interactions and Other behavior categories. Values are means and standard errors of the events in relation to the number of visible animals recorded in 30 s every 5 min over a 19-h observation period (228 scan samples per group). C = Control group, LSA = Low Space Allowance group, HS = Heat Stress group. Asterisks indicate significant differences compared to C group (*p* < 0.05). Figure S1. Relative Humidity, dry and wet temperature recorded during the trials in late spring (HT).
