# Supplementary material for: Effects of Reduced Space Allowance and Heat Stress on Behavior and Eye Temperature in Unweaned Lambs: A Pilot Study
Source: Animals (Basel). 2021 Dec 5;11(12):3464. doi: 10.3390/ani11123464 (PMC8698074; doi:10.3390/ani11123464)

Supplementary material

Table S1. Effect of group and time on behavioural variables included in the Activity and lying position category. Values are means and standard errors of the proportion of visible animals engaging each behaviour collected every 5 minutes over a 19-h observation period (228 scan samples per group). C= Control group in thermoneutral zone TNZ; LSA = Low Space Allowance group in thermoneutral zone TNZ; HS = Heat Stress group. Asterisks indicate significant differences compared to the C group (*p* <0.05).

**Variable**	**Group**			***p*-value**	
	**C (27 cm²/lamb, TNZ)**	**LSA (20 cm²/lamb, TNZ)**	**HS (27 cm²/lamb, heat stress)**	**Group**	**Time**
**Sternal recumbency, head up (%)**	31.48±0.98	36.09*±1.43	22.72*±1.10	<0.001	.969
**Sternal recumbency, head down (%)**	8.08±0.56	5.40*±0.51	5.65*±0.51	<0.001	.576
**Sternocostal recumbency, head up (%)**	14.29±0.75	4.92*±0.32	7.82*±0.52	<0.001	.168
**Sternocostal recumbency, head down (%)**	3.66±0.34	1.25*±0.17	1.65*±0.20	<0.001	.600
**Lateral recumbency**	0.34±0.10	0.09*±0.03	0.04*±0.04	<0.001	.463
**Standing, Suckling /attempt to (%)**	10.08±0.81	10.68*±0.62	8.04*±0.83	<0.001	.041
**Standing, Eating /attempt to (%)**	5.42±0.99	6.90*±1.28	3.88*±1.11	<0.001	<0.001
**Standing, still (%)**	16.12±0.88	21.87*±1.14	37.72*±1.45	<0.001	.911
**Standing, walking (%)**	10.22±0.60	12.67*±0.61	12.61*±0.65	<0.001	.386
**Standing, Playing (%)**	0.54±0.14	0.06±0.04	0.00±	NC	NC

NC= not calculable

Table S2. Effect of group and time on behavioural variables included in the Interactions and Other behaviour categories. Values are means and standard errors of the events in relation to the number of visible animals recorded in 30 seconds every 5 minutes over a 19-h observation period (228 scan samples per group). C = Control group in thermoneutral zone (TNZ); LSA = Low Space Allowance group in thermoneutral zone (TNZ); HS = Heat Stress group. Asterisks indicate significant differences compared to the C group (p <0.05).

Variable	Group			*p*-value	
	C (27 cm²/lamb, TNZ)	LSA (20 cm²/lamb, TNZ)	HS (27 cm²/lamb, heat stress)	Group	Time (h)
**Aggressive interaction when lying (%)**	2.24±0.21	2.80*±0.22	3.03*±0.24	<0.001	.378
**Aggressive interactions when active (%)**	8.63±0.78	8.10*±0.56	7.43*±0.77	<0.001	<0.001
**Non aggressive interaction (%)**	5.96±0.38	3.51*±0.23	6.64*±0.37	<0.001	.154
**Trumpling (%)**	5.06±0.42	5.48*±0.34	3.01*±0.27	<0.001	.838
**Drinking (%)**	0.18±0.07	0.28±0.06	0.33*±0.08	<0.001	.044
**Stretching (%)**	0.37±0.08	0.04*±0.02	0.26*±0.08	<0.001	<0.001
**Shaking (%)**	0.73±0.12	0.68*±0.10	1.01*±0.16	<0.001	.007
**Self-grooming (%)**	2.59±0.22	1.56*±0.14	2.18*±0.21	<0.001	.654
**Stereotypies (%)**	0.52±0.10	1.36*±0.15	0.44*±0.09	<0.001	.940

Figure S1: Relative Humidity, dry and wet temperatures recorded during the trials in late spring (Heat stress).

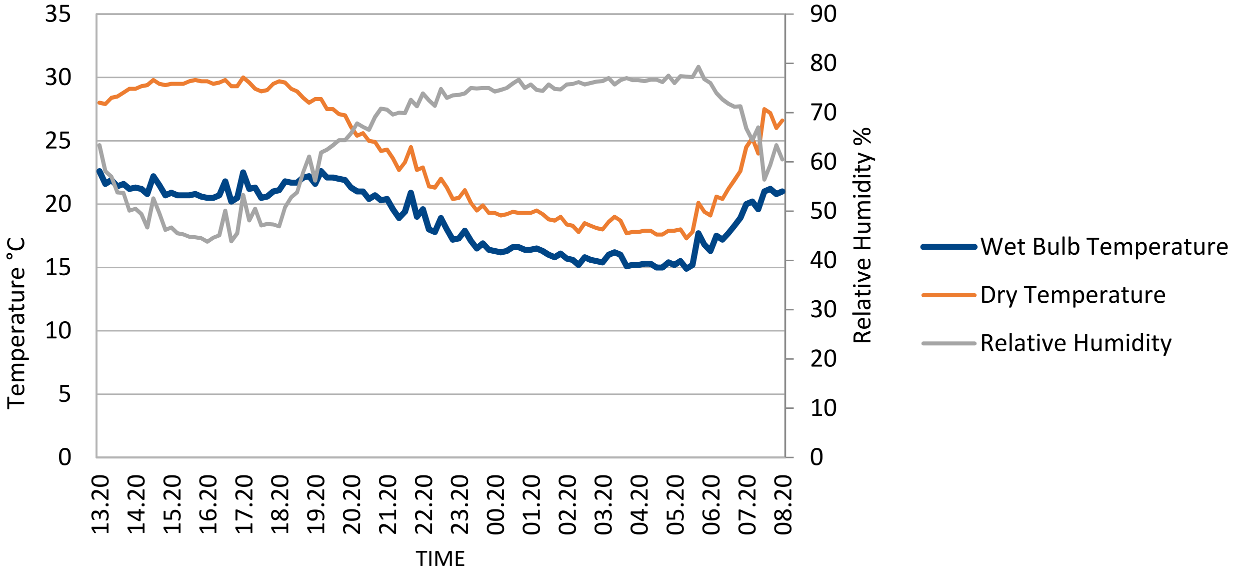

Supplement: Supplementary file 1 [file animals-11-03464-s001.zip › animals-1483841-supplementary.pdf]
